# Editorial: Insights in public health education and promotion: 2022

**DOI:** 10.3389/fpubh.2023.1280357

**Published:** 2023-10-03

**Authors:** Harshad Thakur, Allen C. Meadors

**Affiliations:** ^1^School of Health Systems Studies, Tata Institute of Social Sciences, Deonar, Mumbai, India; ^2^Independent Researcher, Seven Lakes, NC, United States

**Keywords:** public health, health education, health promotion, health sciences, insights, primary care

## 1. Introduction

The discipline of public health received much-needed attention with the pandemic of COVID-19 (1, 2). However, current public health education still needs to be expanded to traditional teaching models that balance theory and practice. With time, public health is also trying to integrate new technologies, especially in education and promotion (3). Primary care and public health comprise the backbone of health systems, but their divergence has produced two groups of practitioners, either focused on individual health or population/public health (4). Public health education is a precursor to public health practice and essential to one's foundational knowledge and skillset. Thus, theoretical solid groundings are critical in public health education (5).

The world is now in the third decade of the 21st Century. The achievements in public health made by scientists have been exceptional, especially in the last few years, leading to significant advancements in the fast-growing field of Public Health Education and Promotion (6). It is essential to highlight the latest advancements in public health science and to shed light on the progress made in the past decade. In addition, its future challenges needs to be identified to provide a thorough overview of the status of the art of the Public Health Education and Promotion field.

For this Research Topic, we received all original research articles. They cover various topics, and each article has a unique study design. Authors were encouraged to identify the most significant challenges in the sub-disciplines and how to address those challenges. Out of 24 submitted manuscripts, 17 articles were selected for publication after a rigorous review. The majority of the articles, i.e., 10, are from China. As China's need for global health capacity grows amid a rapidly shrinking population of younger citizens, strategic investments in transnational public and global health programs may be of increasing value (7). Later, it is followed by two articles from the USA and one each from Ethiopia, Saudi Arabia, UAE, and Indonesia. One article was not country-specific though the authors were from Qatar and Australia.

The articles cover various public health-related subjects related to diseases and subjects like Reproductive issues, Thyroid diseases, Cancer, Drug abuse, Organ transplants, and others. Most article research findings have also addressed medicine/public health curricula, educational content improvement for different age groups, the performance of public health degree programs, and education to strengthen research capabilities.

Here, the author's works are summarized according to the study design.

## 2. Contributions according to study designs

### 2.1. Reviews

In Indonesian schools (both primary and secondary), reproductive health education is integrated into various subjects, including Science, Biology, Sports, and Health Education. Diarsvitri and Utomo conducted a qualitative study comparing the accuracy of the material related to reproductive health education to scientific evidence published in medical journals or medical textbooks. This study was done through a literature review and content analysis of School books of 5 to 12-year age groups. It was found that the schoolbooks were used as per Indonesia's 2006 minimum standard requirements of the subject matter curriculum. Still, the current provision for equipping young Indonesians with comprehensive reproductive health knowledge is inadequate. Schoolbooks must promote healthy lifestyles, prevent high-risk sexual behaviors, encourage openness and discussion about reproductive health in the family, improve self-confidence to refuse and avoid sexual harassment, encourage positive sexual behaviors, and increase awareness for treatment-seeking behavior.

A global video-sharing social media app—TikTok, provides information on Thyroid cancer (TC) which is becoming an increasing public health problem worldwide. However, the information quality of these videos still needs to be discovered. A search of TikTok was performed by Wang et al. with the term “thyroid neoplasm” and “thyroid cancer” in Chinese. The videos included were independently assessed using six predefined questions for content scores and DISCERN (a scale used to judge information quality). The VPI (Video Popularity Index) was calculated. Correlation analysis was performed among duration, presence of animation, VPI, DISCERN scores, and content scores. A total of 56 videos were included, of which 49 were uploaded by physicians, four by health organizations, and three by hospitals. It was seen that 43 videos were real content videos, and 13 were animated. The overall quality of the videos was satisfactory, and it varied greatly depending on the source type. Patients should take proper precautions when using TikTok as a source of TC-related information.

The USA is experiencing exponential growth in overdose fatalities over the past four decades. More than 22 million people in the USA live with a substance use disorder (SUD). The USA Cooperative Extension System (Extension) is recognized as an essential partner in addressing SUD in the communities. The scoping review by Hagaman et al. was performed to identify the range of Extension activities aimed at mediating substance misuse. The authors utilized the PRISMA-SCR model to complete this scoping review in February–July 2022. The scoping review covers a search of peer-reviewed databases, Extension websites for each state and the USA territory, and the utilization of a web search engine. Eighty-seven records meeting the inclusion criteria were included—seven peer-reviewed articles and 80 results from the gray literature. Additional 11 ROTA grantees responded to requests. It was seen that Extension had scaled multiple efforts to address SUD operating through a loose confederation of organizations. The volume of effort is significant. However, implementation at the community level has been slow, and there are significant opportunities for local adoption of evidence-based practices to mitigate SUD.

Applied practice experiences are essential to the Masters of Public Health (MPH) curriculum. The study by Pham et al. examined students' perspectives on the skills and expertise they developed in an MPH course offering applied practice opportunities. From 2008 to 2018, a total of 236 students took the course, and 104 gave their consent. The reflection essays were de-identified and analyzed using a rapid qualitative analysis approach. The essays addressed students' learning experiences and the application of the competencies for MPH programs set by the CEPH (Council for Education in Public Health). The critical lessons by each cohort of students were identified through deductive and inductive analytical lenses. Semi-structured guides and matrixes for essay analysis were created using assignment instructions and CEPH competencies. It was seen that the applied practice experience served as a valuable tool for knowledge and skills acquisition.

It also served as an opportunity for students to engage with the unique organizational cultures of their respective community partners and to deepen their understanding of the complexities of conducting meaningful community-engaged research. This study demonstrates the utility of analyzing students' critical self-reflection to explore learning experiences when training future public health professionals. The findings will be helpful to educators in designing future applied practice experiences.

### 2.2. Cross-sectional studies

The incidence of thyroid diseases has tripled globally in the last three decades, and the prevalence is also rising rapidly, irrespective of gender and genetics. The study by Alhazmi et al. was done to assess the knowledge, awareness of risk factors, and perceptions of thyroid disease among the Saudi Community in Saudi Arabia. An online cross-sectional study was conducted between November 2021 to January 2022 among 724 adult residents (18–50 years) living in Saudi Arabia. Saudi adults reported varying knowledge and perceptions of thyroid disease. Previous knowledge of the thyroid was found to be significantly associated with the current knowledge score. Educating people about this rising disease is essential.

Global contraceptive coverage has increased significantly. Still, high rates of unintended pregnancy are seen globally. A comparative analysis of KAP (Knowledge, Attitude, and Practice) of the Sexual and Reproductive Health (SRH) of both partners will be helpful. Liu et al. conducted a questionnaire survey of people (18–45 years age group) with unintended pregnancies, including women and their male partners (1,275 pairs) from October 2017 to October 2021. The study shows that unintended pregnancy occurs mainly in young people. The risk factors for not taking contraceptive measures are the low education background, the younger age of first sexual intercourse, and the lack of discussion of contraception between partners. Men's better knowledge and contraceptive practices than female partners, and poor male contraceptive knowledge and attitudes may lead to a higher risk of harmful contraceptive practices. The results suggest that male KAP is vital in promoting contraceptive use and reducing unintended pregnancy.

Life form and body composition may affect the health of college students. The study by Lin and Liu explores the relationship between the effects of health behavior and sports participation on 1,200 female college students' body mass index and healthy-promoting lifestyle using the questionnaire method and bioelectrical resistance measurement. Among female college students, there is generally a lack of sleep and leisure activities, a low proportion of regular fitness habits, a high number of snacks, and a high average daily online time. The overweight and body fat rates of female college students are also generally too high, and the standard rate of muscle weight is generally too low. Their health-promoting lifestyle has the highest score of self-realization, followed by interpersonal support, and the worst behavior of sports participation. Among older college students, sports participation and overall health-promotion behavior s quite worse. Those with regular exercise habits have a lower proportion of overweight and high body fat rates.

A study by Ma H. et al. was conducted to investigate the kidney transplantation knowledge of KT (Kidney Transplant) candidates and recipients and explore the related influencing factors. 170 KT candidates and 270 KT recipients were investigated from March to July 2022 in two tertiary and Grade A hospitals in Hunan Province, China, using the Kidney Transplant Understanding Tool (K-TUT). It is seen that the knowledge level of KT candidates and recipients could be more optimistic. Healthcare providers need to pay more attention to the health education of this population.

In China, the organ transplantation sector is facing a severe shortage of donors. The study was conducted by Chen et al. to understand young people's perceptions and attitudes toward organ donation and the factors that influence them and can positively impact the promotion of organ donation. Information was obtained through 501 valid questionnaires from the target group. It is seen that the young people knew about organ donation but needed a higher depth of awareness. The household registration type, education level, and religious affiliation are significantly associated with people's willingness to donate. The correct understanding of the organ donation process, the supportive environment for organ donation in society, and laws and regulations will influence people's willingness to donate.

The need for skilled medical practitioners in outbreak investigations and public health was demonstrated during the COVID-19 pandemic. The College of Medicine and Health Sciences at the United Arab Emirates University (UAEU) introduced a clerkship in public health. This consists of theoretical and practical sessions for 5th-year medical students in 2015. The study by Rahma et al. aims to explore the students' satisfaction with the public health clerkship, which is crucial for assessing and reforming the taught curriculum. A post-evaluation cross-sectional study was conducted from 2015–2022 via an online university system, and 174 students participated. It is seen that the medical students at the UAEU were satisfied with the activities and delivery of the public health clerkship. Conducting needs assessment and proposal writing gave them the knowledge, skills, and confidence to conduct research in their career. These findings may help and support other institutes to plan and develop a clerkship in public health.

As a convenient and promising care model, the public has gradually accepted community-based senior care. However, community services developed to facilitate older adults often need to achieve the expected effect. A study by Ma W. et al. further developed an extended Anderson behavior model by incorporating social psychological factors and vertical and horizontal fairness perceptions. The study used data from a survey of 322 urban area seniors in Shaanxi Province. The results showed that factors influencing older adults' satisfaction with service categories differ. Moreover, with the addition of the social psychological factors, it is observed that the vertical fairness perception of the survey respondents affected their satisfaction with senior care services significantly more than the horizontal fairness perception.

Population knowledge and attitudes toward Obstructive Sleep Apnea syndrome (OSA) are critical to public health initiatives to overcome the disease. Healthcare education is an appropriate approach to expediting the process of building active medical practice models in the public. Pan et al. in their study, aimed to assess the level of KAP regarding OSA and healthcare education demand among the Chinese general population. A cross-sectional survey was performed online via Wenjuanxing in mainland China between February and March 2022. The study enrolled 1,507 respondents, aged 18 to 68 years old. The findings indicated that even the higher educated and urban populations in mainland China needed more knowledge about positive attitudes toward and practices regarding OSA. They showed an urgent demand for health care education. A particular emphasis should be placed on appropriating population demand for health care education and promoting the benefits of active medical practice models in sleep medicine.

### 2.3. Secondary data (ecological study)

Public health education is essential for managing health risks. The study by Gao et al. empirically analyzed the effect of public health education on people's demand for commercial health insurance. The research is based on panel data from 31 provinces in China from 2009 to 2019. It is observed that public health education significantly increases people's demand for commercial health insurance. This effect remains significant when considering endogeneity and robustness. It is also seen that health literacy, health risk perceptions, and health risk attitudes cause the increased demand for commercial health insurance. The effect of health education on promoting people's demand for commercial health insurance is more evident in regions with high levels of urbanization, the proportion of men, education, medical resources, economic development, and social medical insurance coverage.

### 2.4. Retrospective cohort study

Among PLHIV (People Living with the Human Immunodeficiency Virus), Opportunistic infections (OIs) are the leading cause of morbidity and mortality. Nevertheless, there are few robust recent data on the rates of OIs and the risk factors contributing to their occurrence. Woldegeorgis et al. sought to determine the incidence of OIs and identify predictors among adolescents and adults after initiating Anti-Retroviral therapy (ART) in Ethiopia. A retrospective cohort study design was employed. The study population was 515 adolescents and adults who initiated ART between 1 January 2012 and 31 December 2021. The rate of OIs after the initiation of ART was relatively high. Moreover, being female, mild malnutrition, not taking ART, poor adherence to ART, and advanced HIV disease at presentation increased the hazards of developing OIs. Adherence counseling and public awareness can help improve this.

### 2.5. Quasi-experimental study (community trial)

The Chinese government released a national health education program in impoverished counties to promote health literacy among rural populations in 2018. Under this, an integrated health education program was implemented in Yunnan province. This included additional culturally sensitive educational components for the severely impoverished prefectures. Li et al. examined the differential effects of the health education program models on health literacy outcomes among 15-69-year-old residents in poverty-stricken areas. A quasi-experimental design was conducted with two arms. It included surveys at baseline (October 2019) and endline (June 2021) to collect individual-level health information, including the Chinese Resident Health Literacy Scale. The experimental group received the national health education program with the additional Yunnan-specific program, and the control group received only the national program. The findings highlight the importance of incorporating non-verbal visual aids and culturally-sensitive media tools in health literacy education to address healthy lifestyles and the living contexts of the populations in poverty-stricken areas.

### 2.6. Development of tools

The Sugar-Sweetened Media Literacy Scale (SSM-ML) has been shown to significantly assess the US population's SSB (Sugar-Sweetened Beverage) calorie intake. Long and Yoon conducted a cross-sectional study from September to November 2021 to describe the psychometric properties of the revised Chinese version of the SSB-ML (C-SSB-ML) and evaluate its validity and reliability. The results from 975 undergraduates at two of China's most prominent universities showed that the C- SSB-ML criterion-related validity was positively associated with the e-Health Literacy Scale (eHEALS). The findings provide evidence for a valid and reliable tool that can be used to assess sugar-sweetened media literacy in Chinese undergraduates. They will help organizations leverage media literacy in strategy formulation to ensure SSB intake is controlled as much as possible through practical efforts.

Selective biomedical and behavioral approaches still dominate health promotion practice. This is insufficient to reduce health inequities which are quite high due to the inequitable distribution of structural and systemic privilege and power. The RLCHPM (Red Lotus Critical Health Promotion Model) was developed to enhance critical practice. It includes values and principles that the health practitioners will be able to use to critically reflect on health promotion practice. Existing quality assessment tools primarily focus on technical aspects of practice and not on the underpinning values and principles. The purpose of the tool should be to support the reorientation of health promotion practice toward a more critical approach. A project by O'Hara and Taylor aimed to develop a quality assessment tool to support critical reflection using the values and principles of critical health promotion. Critical Systems Heuristics was used as the theoretical framework to develop the quality assessment tool. The pilot testing of the tool was done on nine graduate public health students in 2022. The Quality Assessment Tool for Critical Health Promotion Practice (QATCHEPP) includes ten values and associated principles. It provides theory-based heuristic support for practitioners to use critical reflection to assess the extent to which practice aligns with critical health promotion. The QATCHEPP can be used as part of the RLCHPM or as an independent quality assessment tool to support health promotion orientation toward critical practice. This is essential to ensure that health promotion practice enhances health equity.

## 3. Conclusion

The above studies provide insight into university medical and health science courses and have diverse contributions. This Research Topic is expected to inspire, inform and guide researchers in the field. The Research Topic reiterates the importance of the development of public health education considering local factors like social characteristics, demographic variables, and others. It also highlights the necessity of developing proper tools, educational material, and regular monitoring to sustain educational and promotional initiatives in the vast field of public health.

A new era of computer-assisted education has been opened by the introduction of AI in education has opened. It also brings new possibilities for teaching and learning in public health education (3). The importance of using the latest technological developments for improving public health activities is highlighted and cannot be undermined.

## Author contributions

HT: Conceptualization, Writing—original draft, Writing—review and editing. AM: Conceptualization, Writing—review and editing.
